# Synchronous precessional motion of multiple domain walls in a ferromagnetic nanowire by perpendicular field pulses

**DOI:** 10.1038/ncomms4429

**Published:** 2014-03-24

**Authors:** June-Seo Kim, Mohamad-Assaad Mawass, André Bisig, Benjamin Krüger, Robert M. Reeve, Tomek Schulz, Felix Büttner, Jungbum Yoon, Chun-Yeol You, Markus Weigand, Hermann Stoll, Gisela Schütz, Henk J. M. Swagten, Bert Koopmans, Stefan Eisebitt, Mathias Kläui

**Affiliations:** 1 Institut für Physik, Johannes Gutenberg-Universität Mainz, 55099 Mainz, Germany; 2 Department of Applied Physics, Center for NanoMaterials, Eindhoven University of Technology, PO Box 513, 5600 MB Eindhoven, The Netherlands; 3 Max Planck Institute for Intelligent Systems, Heisenbergstrasse 3, 70569 Stuttgart, Germany; 4 Institut für Optik und Atomare Physik, Technische Universität Berlin, Straße des 17. Juni 135, 10623 Berlin, Germany; 5 Department of Physics, Inha University, Incheon 402-751, Republic of Korea; 6 Helmholtz-Zentrum Berlin für Materialien und Energie GmbH, Hahn-Meitner-Platz 1, 14109 Berlin, Germany

## Abstract

Magnetic storage and logic devices based on magnetic domain wall motion rely on the precise and synchronous displacement of multiple domain walls. The conventional approach using magnetic fields does not allow for the synchronous motion of multiple domains. As an alternative method, synchronous current-induced domain wall motion was studied, but the required high-current densities prevent widespread use in devices. Here we demonstrate a radically different approach: we use out-of-plane magnetic field pulses to move in-plane domains, thus combining field-induced magnetization dynamics with the ability to move neighbouring domain walls in the same direction. Micromagnetic simulations suggest that synchronous permanent displacement of multiple magnetic walls can be achieved by using transverse domain walls with identical chirality combined with regular pinning sites and an asymmetric pulse. By performing scanning transmission X-ray microscopy, we are able to experimentally demonstrate in-plane magnetized domain wall motion due to out-of-plane magnetic field pulses.

## Abstract (web summary)

Magnetic domain walls could form the basis for information technology with high storage density, but require comparatively high current densities to be moved by spin torque. Here, the authors demonstrate a radically different approach with perpendicular magnetic field pulses moving domain walls synchronously.

## Graphical abstract

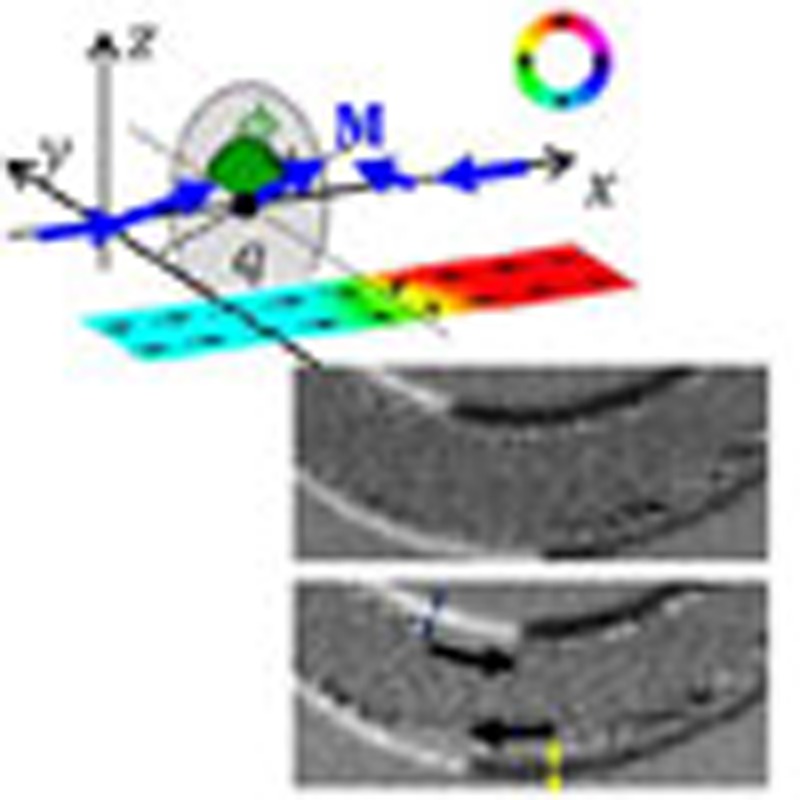

Recently, a broad range of devices based on domain wall (DW) motion has been proposed, including high-density data storage, logic and sensing devices^1^,^2^,^3^,^4^,^5^. Magnetic field-driven DW motion is well established with high DW velocities^6^,^7^,^8^ and allows for non-contact writing without electrical connections to the sample, making it very attractive for many applications. However, the conventional approach of using external magnetic fields parallel to the spin orientation in the domains has been plagued by the seemingly insurmountable problem that the magnetic fields enlarge or shrink domains and eventually lead to a collapse of the domains^9^. Moreover, it is difficult to generate the homogenous in-plane magnetic fields required for the DW motion in the most commonly used in-plane-magnetized permalloy nanowires owing to the lateral geometry of field-generating strip lines. Suggested approaches using in-plane fields and local actuation of DWs lead to slow slip-stick motion and very complex device architecture^10^, which has not been realized because of the complexity and is not deemed very useful for realistic devices. For materials with out-of-plane (OOP) magnetization, it has been proposed to engineer a DW ratchet^11^ to move all DWs in the same direction along a loop by applying alternating fields, but again this entails delicate engineering and this approach does not work in the case of in-plane-magnetized structures.

As an alternative method to manipulate DWs, current-induced DW motion due to the spin torque effect^12^,^13^,^14^ allows for synchronous motion of multiple domains and DWs independent of the spin orientation in the domains and thus forms an alternative way to efficiently manipulate the magnetization configuration in potential novel devices. However, the necessary high currents cause problems, such as Joule heating owing to Ohmic losses^15^,^16^. Furthermore, additional contributions from Rashba and spin Hall effects lead to even more complicated spin dynamics^17^,^18^,^19^. Thus, no device has been presented so far that allows for equally high DW velocities as field-induced motion at realistically low-current densities, rendering this approach challenging for real devices.

Improvements to enhance the DW velocity and push the Walker breakdown to higher fields and currents have been devised by applying additional constant magnetic fields perpendicular to the magnetic wire^8^,^20^,^21^. For symmetry reasons, one expects that these fields do not lead to DW motion themselves and thus cannot provide synchronous motion of multiple DWs for in-plane-magnetized systems. This means a radically different approach is needed as a paradigm shift to render field-induced switching suitable for multiple-DW-based devices. In this paper, we analytically and numerically show that a transverse DW (TW) can be moved by an OOP field pulse. The direction of the TW displacement depends on the field direction and the chirality of the TW. The one-dimensional collective-coordinates model can explain that TW displacement only occurs on the timescale of the non-equilibrium dynamics. Finally, experimental observations of the TW motions are in good agreement with our numerical and analytical results.

## Results

### Theory

As an alternative to current-induced DW motion, we introduce a novel mechanism to move DWs by magnetic fields with the field direction being perpendicular to the in-plane magnetization of the domains and DWs. Such dynamic OOP magnetic field pulses can be generated by electric currents passing through microstrip lines adjacent to the nanowire. Here we extend the one-dimensional collective coordinates model to include the torque generated by the OOP field (which we will show to resemble the effect of the adiabatic spin transfer torque (STT)) and explain how dynamic OOP field pulses can be employed to move DWs. We start with the most general description of the magnetization dynamics induced by fields and spin-polarized currents, governed by the Landau–Lifshitz Gilbert equation^22^,^23^,^24^


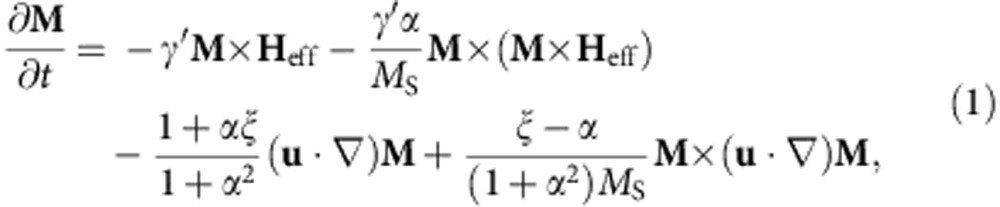


where γ′=γ/(1+*α*^2^) is the gyromagnetic ratio (*γ*=2.211 × 10^5^ m(As)^−1^), **M** is the local magnetization, *M*_S_=|**M**|, and *α* is the viscous Gilbert damping parameter. The first two terms describe the damped precession of the magnetization around the effective magnetic field **H**_eff_. The third and the fourth terms describe the angular-momentum transfer from the spin-polarized conduction electrons to the local magnetization^23^, where **u**=**J**_***e***_*P*μ_B_/*eM*_S_ is the spin drift velocity, *P* is the spin polarization of the conduction electrons and **J**_***e***_ is the electron current density^22^,^23^,^25^. The so-called non-adiabatic STT is proportional to the non-adiabaticity constant *ξ* and describes the torque that does not result from the adiabatic spin transfer.

**Figure 1:**
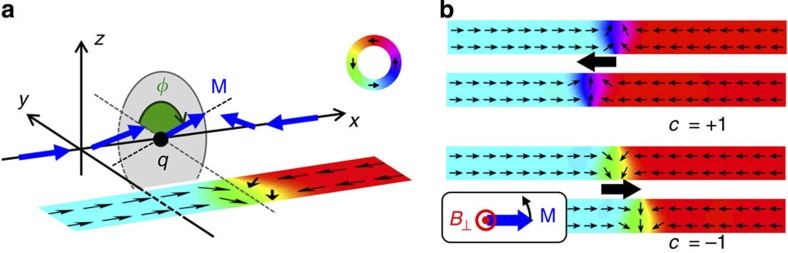
One-dimensional DW model and reversible TW displacement by an OOP field. (**a**) Schematic illustration of the one-dimensional DW geometry to describe an in-plane TW by two collective coordinates, the DW position *q* and the DW angle around the wire axis *φ* (green). (**b**) A head-to-head TW with: positive chirality (*c*=+1) and *p*=+1 (top), and negative chirality (*c*=−1) and *p*=−1 (bottom). For a given field pulse direction, the displacement direction only depends on the chirality with positive (negative) chirality walls being displaced to the left (right) by the application of an OOP field *B*_⊥_=+50 mT along the +*z* direction, which can be understood from the acting torques, as illustrated in the inset.

In the case of in-plane TWs, we can apply the one-dimensional DW model to describe the DW by two collective coordinates, the DW centre position *q* and the angle *φ* (ref. 23) that describes the rotation of the DW around the wire axis, as illustrated in Fig. 1a. By inserting the one-dimensional DW profile into the Landau–Lifshitz Gilbert equation, we obtain two equations that describe the dynamics of the two collective DW coordinates (see Methods)


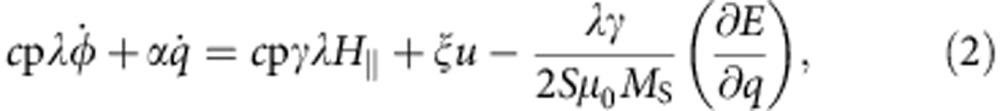



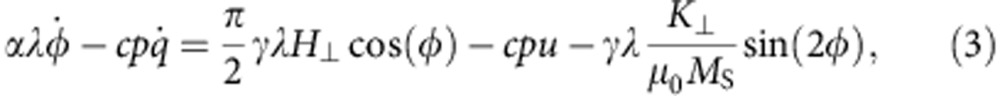


where *λ* is the DW width, *K*_⊥_ is the shape anisotropy perpendicular to the plane that forces the magnetization in the plane of the wire, *B*_|_=μ_0_*H*_|_ and *B*_⊥_=μ_0_*H*_⊥_ are the external driving fields, in-plane parallel (along the *x* direction) and OOP perpendicular (along the *z* direction), respectively. The parameter *p* determines the orientation of the transverse magnetization in the static DW. It distinguishes between a transverse magnetization that points parallel (*p*=+1, Fig. 1b top) or antiparallel (*p*=−1, Fig. 1b bottom) to the *y* axis at the centre of the DW. For a DW in its ground state *p*=cos (*φ*), on the right hand side of equation (2), we can identify the equivalence of the non-adiabatic STT term and the torque due to the in-plane parallel field *H*_|_. The last term in equation (2) describes the position-dependent DW potential energy *E* due to extrinsic pinning, where *S* is the cross-section of the nanowire^26^. The torque arising from the OOP perpendicular field *H*_⊥_, and, more precisely, its direction, depends on the orientation of the magnetization within the TW and hence the term is proportional to cos (*φ*), see equation (3). Therefore, for TWs with cos *φ*≈±1 in nanowires with a strong transverse anisotropy, the torque from the OOP perpendicular field *H*_⊥_ is equivalent to the adiabatic STT. The small angle approximation is reasonably accurate below the Walker breakdown. In the case of in-plane field-driven DW motion, the direction of the displacement depends on the DW type, that is, head-to-head or tail-to-tail, which in our model is given by the DW handedness *cp*=+1 or *cp*=−1, respectively. The chirality *c* defines whether the magnetization rotates counter-clockwise (*c*=+1) or clockwise (*c*=−1) when passing from left to right through the DW. In the ground state, the chirality can be calculated as


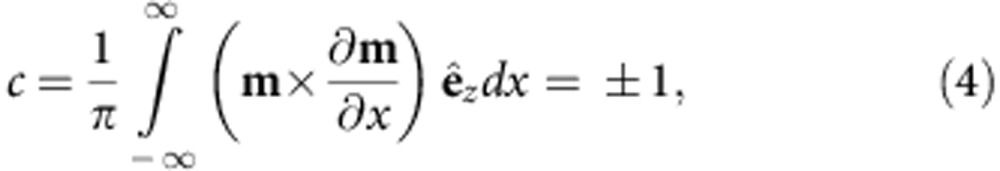


where **m**=**M**/M_S_ is the normalized magnetization and *x* the direction along the wire. For instance, for *φ*=0 (*p*=+1), a head-to-head DW (*cp*=+1) has positive chirality (*c*=+1); see Fig. 1b. In the case of the transient TW dynamics induced by the OOP field, the overall direction of the displacement by OOP fields only depends on the chirality *c* of the DW, as shown in Fig. 1b (see Supplementary Movies 1–3).

To obtain the equation of motion for the quasiparticle DW, we can disentangle the two first-order differential equations of the one-dimensional model to obtain a second-order equation for *q* alone (Supplementary Note 2). For our extended one-dimensional DW model, without a field in the direction of the wire and in the absence of any current, we find


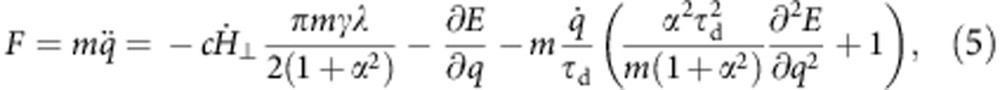


where 
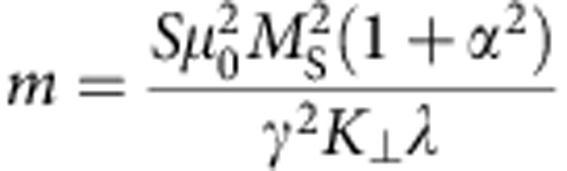
 is the DW mass and 
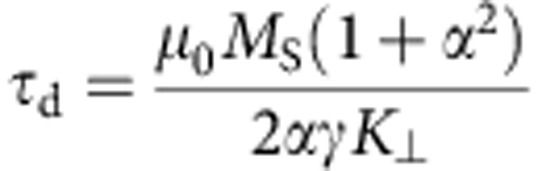
 is the relaxation time. We see that the strength of the force *F* depends on the rise and fall time of the field pulse (namely, on the time derivative 
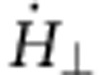
), on the pinning potential landscape 
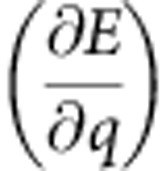
 and on the DW velocity. Intuitively, one would not expect that an OOP field can move the in-plane-magnetized DW since the Zeeman energy does not change when moving a DW in an OOP magnetic field. In particular, continuous motion by static OOP fields is energetically not possible. However, the dynamic forces on the DW described by equation (5) lead to a transient finite-sized displacement during the pulse; after the OOP field is turned off, the DW relaxes back to its original ground state (as detailed in Methods). For instance, in the case of a TW with positive chirality (*c*=+1) with an applied positive OOP field (along +*z* direction), the precession term of the field pulse rotates the magnetization counter-clockwise in the plane similar to precessional switching of single domain particles as shown in ref. 27, thus driving the DW along the wire in −*x* direction, while the damping term leads to an OOP tilting of the spins (along +*z* direction). Eventually, the demagnetization field from this tilt compensates the effect of the applied field and the DW stops. This means that the TW moves a certain distance and, in the absence of pinning, this transient displacement is given by


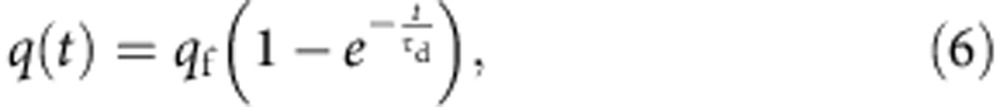


that we obtain directly by solving equation (5). Equation (6) describes the motion from the initial position *q*=0 to the new equilibrium position 
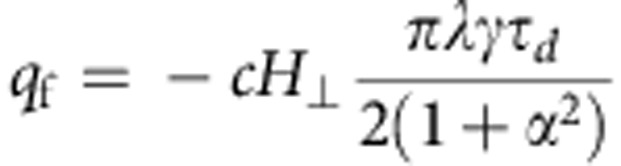
 on application of an OOP field *H*_⊥_. The direction of displacement depends on the chirality *c*, and the magnitude scales inversely with the damping. After switching off the external field, the DW moves back to *q*=0. Such a transient motion is limited to the duration of the non-equilibrium dynamics and is similar in terms of the acting torque directions to the case of purely adiabatic DW motion (*ξ*=0) below the Walker breakdown, where no net DW motion occurs^23^. In the presence of pinning, we can tailor the rise and fall times of the OOP field pulse and thus the resulting force on the DW to depin it from the original pinning potential while keeping it pinned at the next pinning potential and, thus, suppressing the backward motion when the OOF field ramps down. Such a dependence of DW motion in the case of fast changing excitations has been reported recently^28^. As we will show below, a periodic succession of pinning sites can then be used to realize arbitrarily large DW displacements. Experimentally, such a pinning landscape can be geometrically engineered and is additionally always pre-existing, since the edge roughness of the nanowire leads to small changes of the shape anisotropy^28^.

Next, we employ micromagnetic simulations to investigate the performance of this driving mechanism in realistic nanowire geometries. It is of interest how the DW displacement, in particular the distance and the DW velocity, scales with different material parameters, such as the damping parameter.

### Micromagnetic simulations

**Figure 2:**
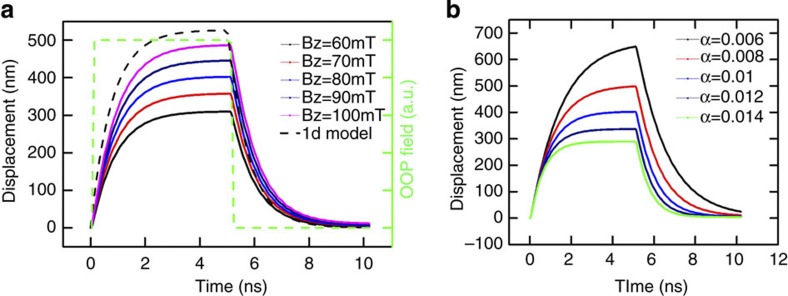
TW displacements by symmetric OOP field pulses. (**a**) TW displacements as a function of time (pulse rise time=100 ps, pulse duration=5 ns and fall time=100 ps) for various OOP field amplitudes. The dashed line indicates the shape of the OOP field pulse with *B*_⊥_=100 mT. The black dashed line shows the TW displacement calculated using the one-dimensional collective coordinate model. (**b**) Dependence of the displacement on the damping constants (*α*=0.006~0.014).

Here we simulate the motion of TWs due to OOP field pulses as a function of field strength and damping strength. For the simulations, a head-to-head TW with negative chirality (*c*=−1) is placed at the centre of a nanowire and a magnetic OOP field pulse (*B*_⊥_=60~100 mT, rise and fall time =100 ps and pulse width=5 ns) is applied along the +*z* direction. Such values are experimentally accessible and lead to a displacement of the TW up to 490 nm. The displacement of the wall *q* as a function of time is plotted in Fig. 2a for variable pulse strength *B*_⊥_. In agreement with our analytical model, the DW position approaches its dynamic equilibrium exponentially, and *q*_f_ scales linearly with the pulse strength. Furthermore, as expected, the TW moves back to the initial position when the OOP field is switched off. The black dashed line in Fig. 2a shows the TW displacement with *B*_⊥_=100 mT calculated by using the one-dimensional DW model, which is in excellent agreement with the results from the micromagnetic simulation. In Fig. 2b we present the TW displacements for various damping constants *α* (0.006<*α*<0.014). Such tuning of the damping in this range can be experimentally realized in wires with DWs, for instance by heavy-ion doping^29^. The maximum displacement *q*_f_ scales inversely with the damping and varies in our simulations between ~300 nm (for *α*=0.014) and ~650 nm (for *α*=0.006). So we see that the interplay of material properties and the excitation provide a powerful handle to tune the DW displacement.

**Figure 3:**
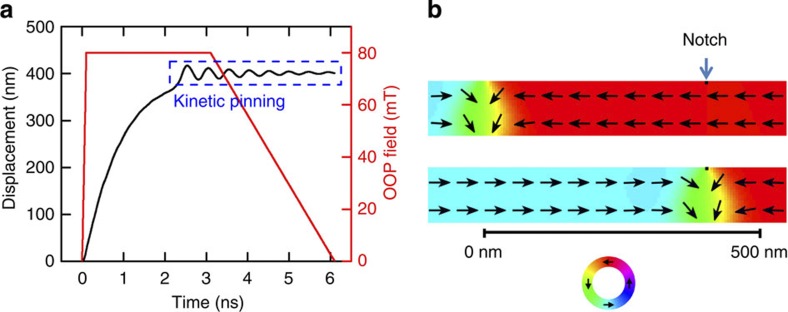
TW motion to a pinning site by an asymmetric OOP field pulse. (**a**) Motion of a TW in a wire with a square notch by an asymmetric OOP field pulse (rise time=100 ps, pulse plateau duration=3 ns and fall time=3 ns). (**b**) The initial (up) and final (down) spin configurations of the TW motion. The notch is located at a position 400 nm to the right of the centre of the nanowire (only part of the wire is shown). A head-to-head TW with down chirality is placed at the centre of the wire. After the wall has moved, it is trapped at the notch position by kinetic pinning. The colour ring indicates the direction of the magnetization.

To suppress the backward motion of the TW when the OOP field is switched off, we introduce an artificial pinning site by tailoring a square notch (5 × 5 nm^2^) at a distance of 400 nm from the initial centre of the nanowire, since the maximum displacement of the TW with *B*_⊥_=80 mT is about 400 nm. For a symmetric field pulse, the torques will be symmetric, so that the DW moves back, but by tuning the rise and fall times, the torques can also be tuned to be asymmetric^30^. Figure 3a shows the displacement of the TW (black line) driven by an asymmetric field pulse (red line). The rise time, pulse width and fall time of the pulse are 100 ps, 3 ns and 3 ns, respectively. As shown in Fig. 3a, a head-to-head TW with negative chirality (*c*=−1) moves along the +*x* direction and as the wall arrives at the notch it becomes kinetically pinned^31^,^32^,^33^, as visible by the damped oscillations of the DW position (black line), in the attractive potential well that the notch generates for the wall^31^,^32^,^33^,^34^,^35^. After 3.1 ns (rise time+pulse width), the OOP field is slowly decreased over a time of 3 ns and the slow fall time leads to small dynamic torques, thus permanently trapping the wall at the pinning site. The snapshots of the TW motion are shown in Fig. 3b. The upper and lower images indicate the initial and final spin configurations of the system, respectively (Supplementary Movie 4). The averaged velocity for this motion is about 65 ms^−1^.

**Figure 4:**
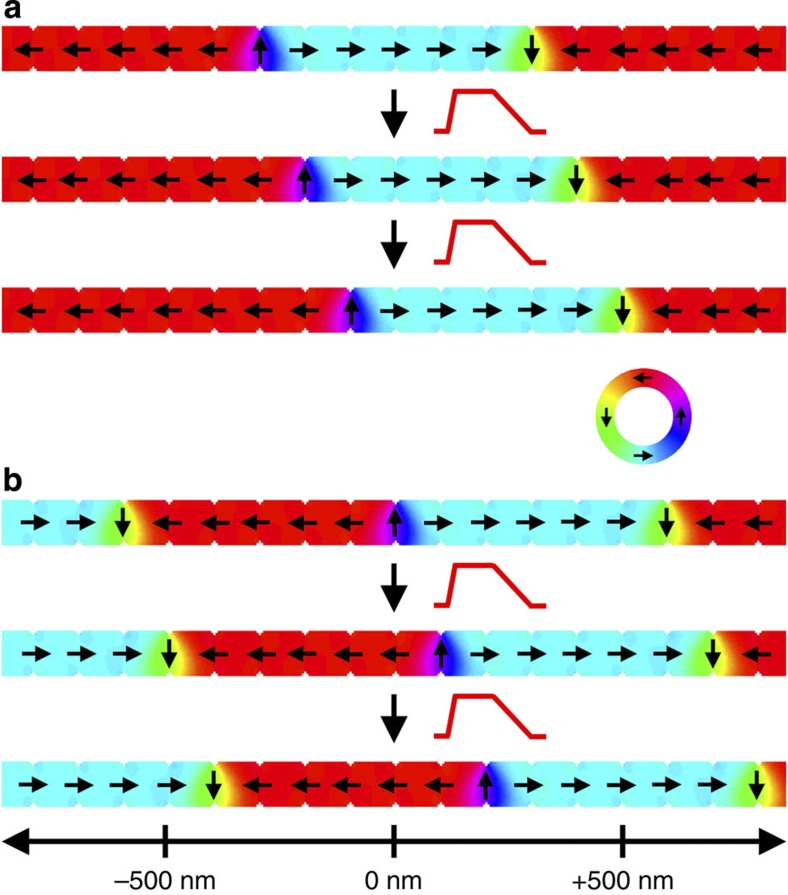
Multiple synchronous TW motion by an OOP field. Successive triangular notches (100 nm spacing) are introduced and TWs are nucleated with 600 nm spacing for both cases of TWs. (**a**) Motion of two TWs with successive OOP pulses *B*_⊥_=110 mT (rise time=100 ps, duration=3 ns and fall time=3 ns). (**b**) Synchronous motion of three TWs with successive OOP field pulses. The colour ring indicates the direction of the magnetization.

A key prerequisite for applications such as the race track memory^3^ or DW logic devices^4^ is the synchronous motion of multiple DWs. Figure 4 shows the synchronous motion of multiple TWs driven by a train of OOP field pulses. The nanowire is 5 μm long, 100 nm wide and 10 nm thick. We have incorporated a realistic geometry of triangular notches (15 nm base × 10 nm height and 100 nm spacing). In Fig. 4 we show the motion of two (a) and three (b) different types of TWs (head-to-head and tail-to-tail), all with identical negative chirality (*c*=−1). To show the independent motion, we set the distance between two successive DWs to 600 nm. We use the same pulse shape as before to depin and move with *B*_⊥_=+110 mT (Supplementary Movies 5 and 6). The TWs move synchronously from one notch to the next notch owing to the OOP field pulse and subsequent pulses always move the walls by a distance of one notch spacing.

### Direct imaging of the DW displacement

**Figure 5:**
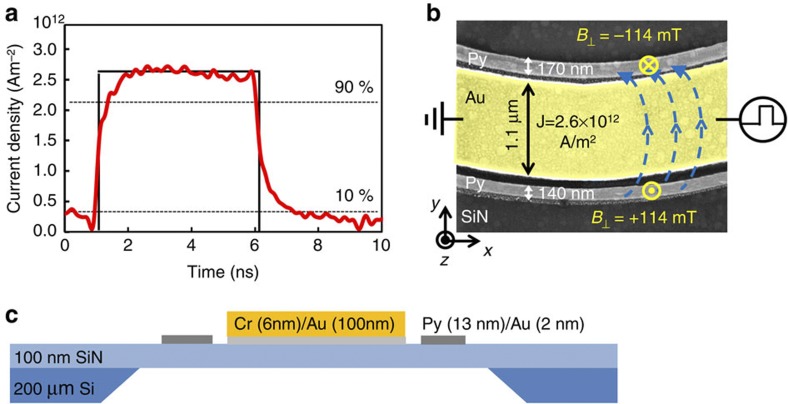
Sample layout and generation of the OOP field. (**a**) The current density *J* in the Au microstrip is plotted as a function of time. The pulse width is 5 ns, the pulse amplitude is *J*=2.6 × 10^12^ Am^−2^ and the pulse is asymmetric, as the rise time of 700 ps is more than 50% faster than the fall time of 1,100 ps. (**b**) False colour scanning electron micrograph of two curved permalloy nanowires fabricated next to a gold microstrip (yellow). The magnetic field, generated by the current pulse injected through the microstrip, is perpendicular to the plane and opposite in direction at the position of the nanowires on top and on the bottom of the microstrip, as indicated by the blue arrows. (**c**) Schematic illustration showing the cross-section of the device on top of a silicon nitride (SiN) membrane.

**Figure 6:**
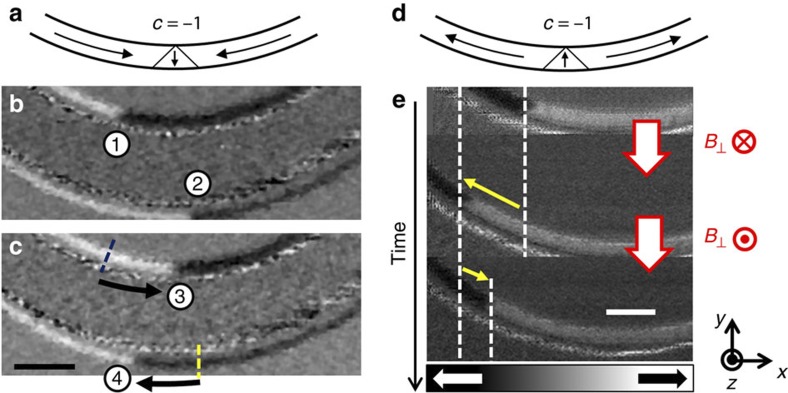
Permanent TW motion induced by an OOP field pulse. (**a**) The initial TW configuration (*p*=1 and *c*=−1) of the two TWs in **b** and **c** is schematically illustrated. (**b**,**c**) Grey scale X-ray magnetic circular dichroism (XMCD)–scanning transmission X-ray microscopy (STXM) images showing the magnetic contrast of the domains and two head-to-head TWs with negative chirality before (**b**) and after (**c**) the injection of five subsequent pulses through the microstrip. The OOP field pulse is positive (negative) on top (at the bottom) of the microstrip, as illustrated in Fig. 5a,b. The upper TW moves a distance of 840±20 nm from position (1) to position (3), while the lower TW travels −940±20 nm from position (2) to position (4). Scale bar, 1 μm. (**d**) The initial TW configuration (*p*=−1 and *c*=−1) of the TW in **e** is schematically illustrated. (**e**) A XMCD–STXM image series showing a tail-to-tail TW with negative chirality before and after the injection of two subsequent and opposite in direction OOP field pulses. The TW travels 680±20 nm to the left (in −*x* direction) after the application of a negative OOP field pulse *B*_⊥_=−114 mT. In contrast, the TW moves 340±20 nm to the right (in +*x* direction) after the application of a positive OOP field pulse *B*_⊥_=+114 mT. In all images, white (black) contrast corresponds to magnetization pointing to the right (left). Scale bar, 500 nm.

Finally, we test the proposed displacement mechanism experimentally. Permalloy half rings (170 nm wide and 13 nm thick) have been fabricated next to a 1.1-μm-wide curved Au microstrip, as shown in Fig. 5b (for details see Methods). After saturation with a uniform magnetic field, two TWs are formed at the centre of the nanowires, as shown in Fig. 6a. The dimensions of the nanowires are chosen such that the TW is the ground state^35^. The DW spin structure was imaged with sub-30 nm spatial resolution scanning transmission X-ray microscopy (see Methods).

To displace the TWs, we applied OOP magnetic field pulses by injecting asymmetric current pulses through the Au microstrip, as illustrated in Fig. 5a,b. The pulse width is 5 ns and the pulse is asymmetric, since the rise time of 700 ps is about 50% faster than the fall time of 1,100 ps. The maximum pulse amplitude is *J*=2.6 × 10^12^ Am^−2^, corresponding to a maximum OOP field of *B*_⊥_=114 mT at the centre of the magnetic nanowire. The positions of two TWs were imaged before and after the application of five successive OOP field pulses. In Fig. 6a, the initial configuration of the two head-to-head TWs is schematically illustrated. The chirality of both DWs is negative (*c*=−1) after nucleation. The DW positions before and after five subsequent applications of OOP field pulses are shown in Fig. 6b and c, respectively. We see that the two opposite field directions result in permanent DW displacements in the two opposite directions for the two DWs; the upper DW having moved a distance of 840±20 nm from position (1) to position (3), while the lower DW travelled −940±20 nm from position (2) to position (4).

To show bidirectional movement, we imaged the DW position before and after the application of two OOP field pulses with opposite sign. The initial TW configuration is shown in Fig. 6d where the chirality of the tail-to-tail TW is negative. In Fig. 6e, a series of three images before and after the application of two subsequent and oppositely directed OOP field pulses is shown. The TW travelled 720±20 nm to the −*x* direction after the application of a negative OOP field pulse *B*_⊥_=−114 mT. In contrast, the TW moved 340±20 nm to the right (in +*x* direction) after the application of a positive OOP field pulse *B*_⊥_=+114 mT. To obtain completely reproducible motion, one needs to make sure that the driving field pulse provides an effective force on the wall that is sufficiently strong to overcome natural pinning. In combination with artificial pinning sites (notches), which are significantly stronger than the natural pinning, this will lead to reproducible motion. In our experimental work, we so far rely on the natural pinning at edge roughness or other defects, which is of course not constant along the wire and therefore it is not surprising that the motion is not always identical for both directions.

## Discussion

Now we discuss the experimentally observed DW displacement and compare it with theory to gauge the applicability of our approach as an alternative to current-induced DW motion for applications.

As visible in Fig. 6, the permanent displacement of DWs by asymmetric OOP field pulses is confirmed by direct imaging experiments. Qualitatively, our observations confirm the predictions of the analytical model and the micromagnetic simulations that the direction of the DW can be controlled by the direction of the OOP field pulse. The measured displacement is insensitive to the spin orientation within the domains, which is a critical prerequisite for the synchronous motion of multiple domains. Furthermore, we have observed the predicted dependence of the direction of the displacement on the TW chirality. When the final position deviates slightly from the initial position, we can attribute this to thermal activation and the non-symmetric potential landscape of the nanowire due to imperfections from the fabrication process, which results in an asymmetric displacement even for identical pulse shapes.

Next, we consider the dependence of the wall displacement on the materials’ parameters and geometry of the magnetic wire to maximize a possible device performance. From equation (6) it is clear that higher field pulses lead to a larger displacement, which is also visible in Fig. 2a. Furthermore, the maximum displacement is directly proportional to the time constant *τ*_d_ of the transient motion. This quantity scales inversely with the damping *α* and inversely with the ratio of transverse anisotropy *K*_⊥_ and saturation magnetization *M*_*S*_. That is, the wall displacement as well as the wall velocity can be increased by using materials with a smaller damping constant (Fig. 2b) or a larger saturation magnetization or by tailoring the wire geometry to decrease the transverse anisotropy. The second important aspect is the sensitivity to edge roughness and other defects. From equation (5) one sees that the effective force acting on the DW quasiparticle scales with the time derivative of the field pulse, showing that tuning the field pulse shape (rise time and fall time) can be used to set both the acting forces as well as the displacement distances and by engineering the pulse shape, the desired displacement can be reached for a given edge roughness, pinning site spacing and pinning site strength.

To be of interest for applications, the efficiency of the OOP field-driven TW motion has further to be compared with current-induced DW motion. Although this precessional TW motion by the OOP field generated by injecting an electric current in Au microstrip is not easy to directly compare with the TW motion due to the STT effect, we can compare energy dissipations for the DW motion using both effects.

First, for the case of the TW motion driven by the OOP field, the experimental TW displacement in a 170-nm wide and 13 nm thick Py nanowire is ~840 nm for a 5-ns pulse in the Au microstrip with 1.1 μm width and 100 nm thickness with a current density of 2.6 × 10^12^ Am^−2^. The simulations from Fig. 4 show that our scheme works well for DWs with a distance of 600 nm. To obtain a displacement of a DW over this distance within 5 ns, we need a current density of 1.7 × 10^12^ Am^−2^ since the maximum displacement scales linearly with the current density. Using a specific resistivity of Au of 2.44 μ Ωcm, we find that the dissipated energy is 22 pJ per moved DW.

For the case of the DW motion driven by the STT effect, we now compare the theoretical and numerical calculations of the DW motion^23^ since many different experimentally measured DW velocities with in-plane-magnetized materials were reported^12^,^13^,^36^,^37^. We assume that the spin polarization is 0.4 (ref. 38) and the non-adiabaticity factor is the same as the Gilbert damping constant. With a saturation magnetization of Py of 8 × 10^5^ Am^−1^ this yields a current density of 4.2 × 10^12^ Am^−2^. With a specific resistivity of Py of about 40 μ Ωcm (ref. 39), the energy for the displacement of one DW is about 46 pJ. The high-current density in the Py wire will increase the temperature of the wire and thus increase the specific resistivity. This will further increase the amount of energy needed for the motion of one wall.

Therefore, the energy dissipation of our system is quite competitive even with the theoretically predicted DW motion by the STT torque on a defect-free nanowire. Further advantages of our system are the following. First, it is possible that the TW can be displaced backwards when the magnetic field switched off (see Fig. 5a). Although the fall time of the OOP field pulse is longer than the rise time, there is indeed a torque with opposite direction, which drives the TW backwards. Therefore, the maximum displacement of the TW might be larger than our observation of 840 nm. Finally, since the precessional torque wants to rotate the TW, this torque does not nucleate an anti-vortex core, which prevents the motion of the DW in the case of current-induced domain wall motion above the Walker breakdown. Thus the field-induced motion does not suffer from the slow velocities obtained for high-current densities resulting from the Walker breakdown.

Note that an energy of tens to hundreds of pJ is used to write for instance FLASH memory, so this can be provided by the complementary metal-oxide-semiconductor.

Another key issue is the cross-talk between individual devices, which can be caused by the OOP field generated by the Au microstrip extending into space. This can affect the ultimate data storage density that can be realized in a racetrack technology. To investigate this cross-talk issue, we simulate the depinning field (not shown) for a wire with the same geometry (width=140 nm and thickness=13 nm). The depinning field from a square notch (5 × 5 nm^2^) representing typical edge roughness is ~60 mT. This field corresponds to the field generated at a distance of 450 nm from the edge of the Au microstrip. Hence, as long as we space the wires at a larger distance, we can prevent significant cross-talk.

In summary, the here presented use of perpendicular field pulses provides the necessary paradigm shift to achieve synchronous DW motion of multiple DWs by magnetic fields. We show that we can move synchronously multiple walls by OOP field pulses, thus providing the necessary functionality for non-volatile DW-based shift register devices. It should be noted that this approach also works for the technologically more relevant OOP-magnetized wires when applying in-plane field pulses, as this just constitutes a rotation in spin space without changing the underlying physics. The direction of the TW displacement depends on the field direction and the chirality (*c*=±1) of the TW and the one-dimensional collective coordinates model shows us that TW displacement only occurs on the timescale of the non-equilibrium dynamics. By applying an asymmetric field pulse and introducing notches as pinning sites, an irreversible motion over distances of hundreds of nm is observed with a high velocity. Finally, experimental observations are in good agreement with our analytical and numerical results, confirming our proposed novel mechanism for DW displacement.

## Methods

### Analytical model

In the one-dimensional model, the total micromagnetic energy *E* is given by the sum of the exchange energy and the shape anisotropies.


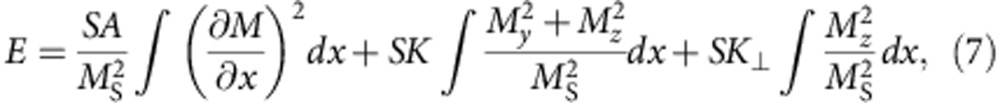


where *S* is the cross-section of the wire and *A* is the exchange constant. *K* and *K*_⊥_ are the anisotropy constant perpendicular to the wire and perpendicular to the plane, respectively.

The first two terms are minimized by a magnetization given by


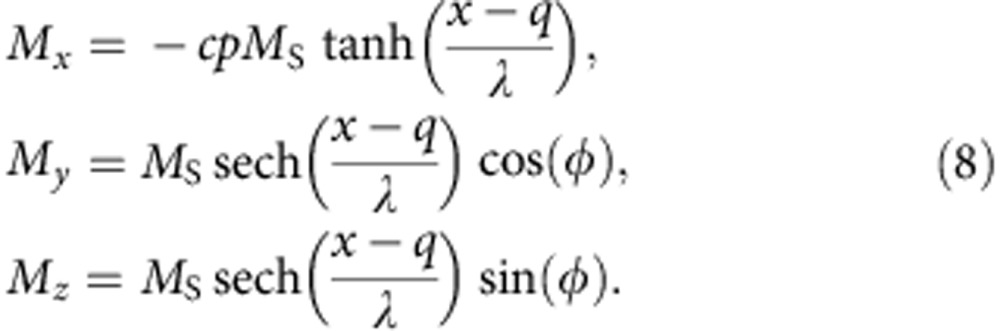


The DW width *λ* is given by 
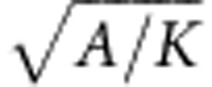
, *q* is the position of the centre of the DW and *φ* is the angle that describes the rotation of the DW around the wire axis. The value *cp* distinguishes between a head-to-head (*cp*=+1) and tail-to-tail (*cp*=−1) DW. In the ground state, the third term in equation (7) forces the DW to rotate in-plane which means sin (*φ*)=0. The parameter *c* determines the rotation of the magnetization in the DW as defined in equation (4).

The magnetization depends on the collective coordinates *q* and *φ* that determine the current state of the wall. The equations of motion of these coordinates are


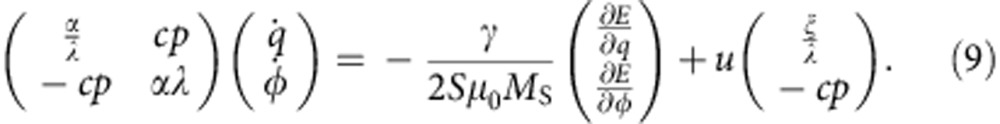


As is, for example, derived in ref. 26. These equations are non-explicit in 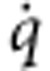 and 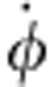. Explicit equations can be calculated by multiplying equation 9 with


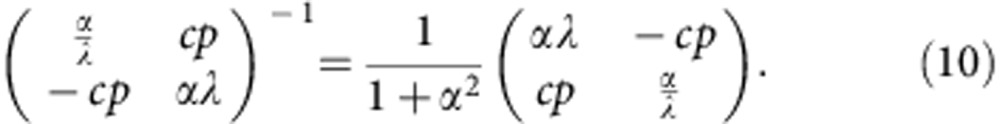


This yields the explicit equation


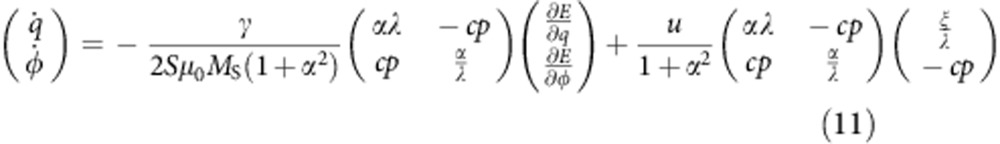


that is, for example, derived in ref. 40.

With an external field, the energy in equation (11) consists of three contributions. A magnetic field that is applied parallel to the wire in *x* direction yields the energy


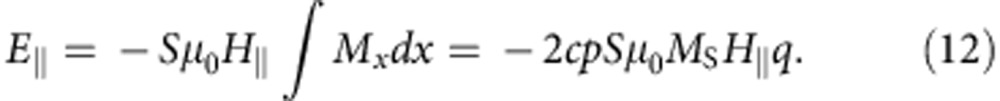


A magnetic field perpendicular to the plane leads to an energy


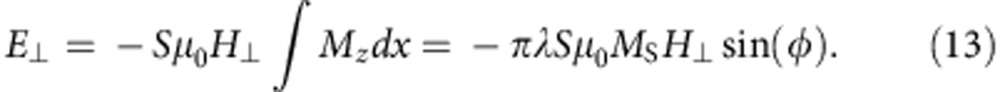


From the energy in equation (7) there is only the third term left as the former ones are independent of *q* and *φ*. The perpendicular anisotropy energy reads


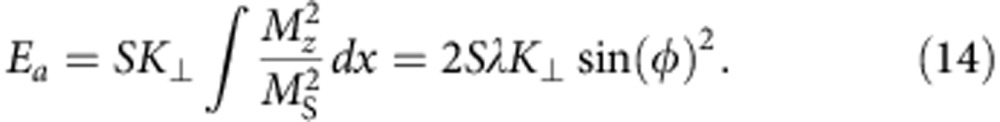


Now we can calculate the sum *E* of these energies and its derivatives with respect to *q* and *φ*. Inserting the derivatives into equation (11) yields


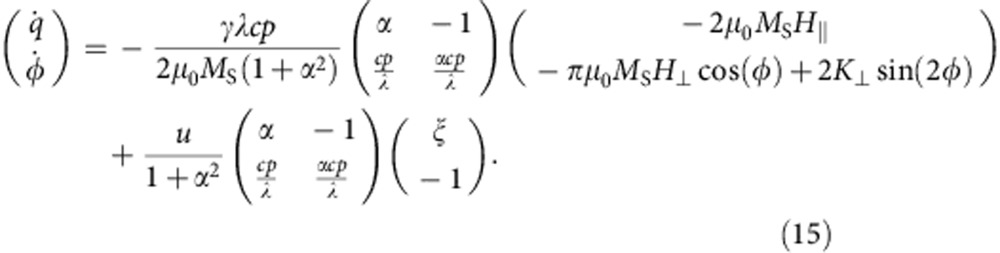


We have numerically calculated the DW displacement under the application of a symmetric OOP field pulse (5 ns width and *B*_⊥_=100 mT), using our extended one-dimensional DW model. We choose realistic parameters for a TW in a 150-nm wide and 10 nm thick permalloy nanowire: saturation magnetization *M*_S_=800 × 10^3^ Am^−1^; transverse anisotropy^40^,^41^ *K*_⊥_=3.7 × 10^5^ Jm^−3^; damping constant *α*=0.01; dynamic DW width^42^ *λ*=30 nm. Initial conditions are *q*_0_=0 and *φ*_0_=0.

### Micromagnetic simulations

The micromagnetic simulations were carried out with the object-oriented micromagnetic framework^43^. We choose a cell size (5 × 5 × thickness nm^3^) below the exchange length and standard material parameters for permalloy are used in the simulations: saturation magnetization *M*_S_=800 × 10^3^ Am^−1^ exchange stiffness *A*=1.3 × 10^−11^ Jm^−1^ and a damping constant *α*=0.01 (0.006<*α*<0.014 for Fig. 2b).

### Experiment

The permalloy Ni_80_Fe_20_(13 nm)/Au(2 nm) half rings were fabricated on top of a 100-nm-thick silicon nitride membrane, next to curved Cr(6 nm)/Au(100 nm) microstrip, as illustrated in Fig. 5. All structures were prepared using electron-beam lithography and lift-off processing. The permalloy was deposited by molecular beam evaporation in ultra high vacuum and the gold was thermally evaporated. The OOP field component of the microstrip decays as *r*^−1^. However, since the permalloy curved nanowire is about 170 nm wide, we can assume a constant perpendicular field with an error of less than ±20%. Furthermore, the generated field is almost homogenous along the magnetic nanowire, owing to the fact that the gold microstrip is placed next to the magnetic nanowire at a constant distance of 40 nm (see Fig. 5). The TW moved in either the left or right direction after the injection of a positive or negative asymmetric burst pulse, respectively. The in-plane component of the magnetization was imaged by scanning transmission X-ray microscopy at the MAXYMUS end station, Helmholtz Zentrum Berlin, BESSY II, Germany, with the sample tilted by 60° with respect to the X-ray beam and magnetic contrast provided through the X-ray magnetic circular dichroism^44^. The data have been recorded at the Ni L_3_ absorption edge (852.7 eV).

## Author contributions

J.-S.K., C.-Y.Y. and M.K. conceived the project; micromagnetic simulations were done by J.-S.K. and data analysis was done by J.-S.K., J.Y., C.-Y.Y., M.-A.M., A.B. and M.K.; M.-A.M. and T.S. fabricated the sample; J.-S.K., C.-Y.Y., M.-A.M., A.B., F.B., B.K., R.R. and M.K. prepared the manuscript; the measurements were done by M.-A.M., A.B., T.S. and F.B.; the technical support for the measurement was provided by M.W. and H.S. The analytical calculations were done by B.K., A.B. and M.K.; G.S., H.S., H.J.M.S., B.K., S.E. and M.K. supervised the project and all authors discussed the results.

## Additional information

**How to cite this article:** Kim, J.-S. *et al.* Synchronous precessional motion of multiple domain walls in a ferromagnetic nanowire by perpendicular field pulses. *Nat. Commun.* 5:3429 doi: 10.1038/ncomms4429 (2014).

## Supplementary Material

Supplementary Notes Supplementary Notes 1-2

Supplementary Movie 1 Head-to-head domain wall motion by an OOP field (c=-1)This movie shows the displacement of the head-to-head domain wall with the chirality (c= -1). The out-of-plane magnetic field is applied with B_z = 100 mT.

Supplementary Movie 2 Head-to-head domain wall motion by an OOP field (c=+1)This movie shows the displacement of the head-to-head domain wall with the chirality (c= +1). The out-of-plane magnetic field is applied with B_z = 100 mT.

Supplementary Movie 3 Tail-to-tail domain wall motion by an OOP field (c=+1)This movie shows the displacement of the tail-to-tail domain wall with the chirality (c= +1). The out-of-plane magnetic field is applied with B_z = 100 mT.

Supplementary Movie 4 Domain wall pinningThis movie shows the irreversible transverse wall displacement with and asymmetric out-of-plane field pulse (rise time = 100 ps, duration = 3 ns, and fall time = 3 ns).

Supplementary Movie 5 Two TW motion by an OOP fieldTwo individual domain walls are moved by two successive asymmetric out-of-plane field pulses (rise time = 100 ps, pulse duration time = 3 ns, fall time = 3 ns, and the amplitude of the out-of-plane field = 110 mT.) In the wire, triangular notches are periodically placed (15 nm base × 10 nm height and 150 nm spacing). A 5 m long, 150 nm wide, and 10 nm thick Py nanowire is used.

Supplementary Movie 6 Three TW motion by an OOP fieldThree individual domain walls are moved by two successive asymmetric out-of-plane field pulses (rise time = 100 ps, pulse duration time = 3 ns, fall time = 3 ns, and the amplitude of the out-of-plane field = 110 mT.) In the wire, triangular notches are periodically placed (15 nm base × 10 nm height and 150 nm spacing).
