# Supplementary material for: Synchronous precessional motion of multiple domain walls in a ferromagnetic nanowire by perpendicular field pulses
Source: Nat Commun. 2014 Mar 24;5:3429. doi: 10.1038/ncomms4429 (PMC4077121; doi:10.1038/ncomms4429)
Supplement: Supplementary Notes — 1-2 [file ncomms4429-s1.pdf]

Supplementary Note 1

From equation (15) in the paper, it becomes obvious that the velocity caused by a field parallel to the wire direction depends on the type of the wall, that is, head-to-head or tail-to-tail. This causes neighboring walls to annihilate each other. The non-adiabatic spin-transfer torque has the same influence on the domain wall as a parallel field while the perpendicular field is similar to the adiabatic spin-transfer torque despite the $\cos(\phi)$. For the adiabatic spin-transfer torque it has already been found by Zhang and Li[1] that a transient displacement of the wall is caused but no continuous motion. We will now show that it is the same for the perpendicular field.

Without a field in the direction of the wire and in the absence of any current, equation (15) in the paper becomes

$$\begin{pmatrix} \dot{q} \\ \dot{\phi} \end{pmatrix} = \frac{\gamma}{2\mu_0 M_\mathrm{S}(1+\alpha^2)} \begin{pmatrix} -cp\lambda \\ \alpha \end{pmatrix} \left[\pi\mu_0 M_\mathrm{S} H_\perp \cos(\phi) - 2K_\perp \sin(2\phi)\right]. \tag{1}$$

For the analytical calculation, we assume that the angle ϕ experiences only small changes compared to the groundstate. For this we express ϕ by the sum

$$\phi = \Delta\phi + \pi\frac{1-p}{2} \tag{2}$$

of the change $\Delta\phi$ and the equilibrium value $\pi(1-p)/2$. Then the equation of motion can be linearized in $\Delta\phi$ and reads

$$\begin{pmatrix} \dot{q} \\ \dot{\Delta\phi} \end{pmatrix} = \frac{\gamma}{2\mu_0 M_\mathrm{S}(1+\alpha^2)} \begin{pmatrix} -cp\lambda \\ \alpha \end{pmatrix} \left(\pi\mu_0 M_s H_\perp p - 4K_\perp \Delta\phi\right). \tag{3}$$

From the second equation we can calculate

$$\Delta\phi = \frac{\pi\mu_0 M_\mathrm{S} H_\perp p}{4K_\perp}\left(1 - e^{-\frac{t}{\tau_\mathrm{d}}}\right). \tag{4}$$

where, we define the damping time

$$\tau_\mathrm{d} = \frac{\mu_0 M_\mathrm{S}(1+\alpha^2)}{2\gamma K_\perp \alpha} \tag{5}$$

in which the free wall slows down to $1/e$ of its initial velocity. From the first equation (3) we get the velocity

$$\dot{q} = -\frac{\pi\gamma\lambda H_\perp c}{2(1+\alpha^2)} e^{-\frac{t}{\tau_\mathrm{d}}} \tag{6}$$

with respect to time. It becomes visible that the domain wall slows down with time. The direction of the wall motion only depends on the direction of the OOP field and on the chirality of the wall. An integration with respect to time yields the displacement

$$q = -\frac{\pi\lambda\mu_0 M_{\mathrm{S}} H_\perp c}{4\alpha K_\perp}\left(1 - e^{-\frac{t}{\tau_{\mathrm{d}}}}\right). \tag{7}$$

It can be seen that this displacement is finite.

Supplementary Note 2

For the calculation of the acceleration of the domain wall we start from the equation of motion

$$\begin{pmatrix} \dot{q} \\ \dot{\phi} \end{pmatrix} = \begin{pmatrix} -\alpha & -\frac{\lambda c p}{\alpha} \\ -\frac{cp}{\lambda} & 1 \end{pmatrix} \begin{pmatrix} \frac{\alpha\tau_d}{m(1+\alpha^2)}\frac{\partial E}{\partial q} \\ \frac{\gamma\alpha\pi p H_\perp}{2(1+\alpha^2)} - \frac{\phi}{\tau_d} \end{pmatrix} \tag{8}$$

that is linearized in ϕ and includes a potential E that depends on the position of the wall. Here, we use the domain wall mass

$$m = \frac{S\mu_0^2 M_{\mathrm{S}}^2 (1+\alpha^2)}{\gamma^2 K_\perp \lambda}. \tag{9}$$

The acceleration of the wall can be determined from a time derivative of the first equation in (8). This reads

$$\ddot{q} = -\frac{\alpha^2\tau_{\mathrm{d}}}{m(1+\alpha^2)}\dot{q}\frac{\partial^2 E}{\partial^2 q} - \frac{\gamma\lambda\pi c\dot{H}_\perp}{2(1+\alpha^2)} + \frac{\lambda c p\dot{\phi}}{\alpha\tau_{\mathrm{d}}}, \tag{10}$$

where $\dot{\phi}$ can be replaced using the second equation in (8) to get

$$\ddot{q} = -\frac{\alpha^2\tau_{\mathrm{d}}}{m(1+\alpha^2)}\dot{q}\frac{\partial^2 E}{\partial^2 q} - \frac{\gamma\lambda\pi c\dot{H}_\perp}{2(1+\alpha^2)} + \frac{\lambda c p}{\alpha\tau_{\mathrm{d}}}\left(-\frac{\alpha c p\tau_{\mathrm{d}}}{\lambda m(1+\alpha^2)}\frac{\partial E}{\partial q} + \frac{\gamma\alpha\pi p H_\perp}{2(1+\alpha^2)} - \frac{\phi}{\tau_{\mathrm{d}}}\right). \tag{11}$$

To get an expression that is independent of the angle ϕ we rewrite this expression as

$$\ddot{q} = -\frac{\alpha^2\tau_{\mathrm{d}}}{m(1+\alpha^2)}\dot{q}\frac{\partial^2 E}{\partial^2 q} - \frac{\gamma\lambda\pi c\dot{H}_\perp}{2(1+\alpha^2)} - \frac{1}{m(1+\alpha^2)}\frac{\partial E}{\partial q} + \frac{1}{\tau_{\mathrm{d}}}\left(\frac{\gamma\lambda\pi c H_\perp}{2(1+\alpha^2)} - \frac{\lambda c p\phi}{\alpha\tau_{\mathrm{d}}}\right). \tag{12}$$

Using the first equation in (8) one obtains

$$\ddot{q} = -\frac{\alpha^2\tau_{\mathrm{d}}}{m(1+\alpha^2)}\dot{q}\frac{\partial^2 E}{\partial^2 q} - \frac{\gamma\lambda\pi c\dot{H}_\perp}{2(1+\alpha^2)} - \frac{1}{m(1+\alpha^2)}\frac{\partial E}{\partial q} - \frac{1}{\tau_{\mathrm{d}}}\left(\dot{q} + \frac{\alpha^2\tau_{\mathrm{d}}}{m(1+\alpha^2)}\frac{\partial E}{\partial q}\right) \tag{13}$$

which is independent of ϕ. By rearranging the terms

$$F = m\ddot{q} = -m\left(\frac{\alpha^2\tau_{\mathrm{d}}^2}{m(1+\alpha^2)}\frac{\partial^2 E}{\partial^2 q} + 1\right)\frac{\dot{q}}{\tau_{\mathrm{d}}} - \frac{m\gamma\lambda\pi c\dot{H}_\perp}{2(1+\alpha^2)} - \frac{\partial E}{\partial q} \tag{14}$$

one finds that the force is given by a velocity dependent damping, an external force that is proportional to the time derivative of the field, and the confining force of the potential.

Supplementary Reference

[1]S. Zhang and Z. Li, *Roles of Nonequilibrium Conduction Electrons on the Magnetization Dynamics of Ferromagnets*. Phys. Rev. Lett. **93**, 127204 (2004).
