# Supplementary figures and images for: Synchronous precessional motion of multiple domain walls in a ferromagnetic nanowire by perpendicular field pulses
Source: Nat Commun. 2014 Mar 24;5:3429. doi: 10.1038/ncomms4429 (PMC4077121; doi:10.1038/ncomms4429)

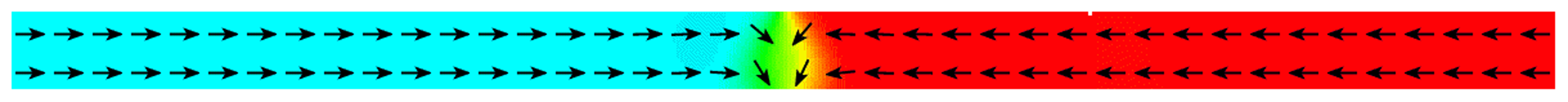

Supplement: Supplementary Movie 4 — Domain wall pinningThis movie shows the irreversible transverse wall displacement with and asymmetric out-of-plane field pulse (rise time = 100 ps, duration = 3 ns, and fall time = 3 ns). [file ncomms4429-s5.tif]
